# Integrating artificial intelligence with circulating tumor DNA for non-small cell lung cancer: opportunities, challenges, and future directions

**DOI:** 10.3389/fmed.2025.1612376

**Published:** 2025-06-11

**Authors:** Nishanth Thalambedu, Mamtha Balla, Barath Prashanth Sivasubramanian, Prasanth Sadaram, Krishna Prathiba Malla, Krishna P. Vasipalli, Sunil Kakadia

**Affiliations:** ^1^Department of Hematology and Oncology, University of Arkansas for Medical Sciences, Little Rock, AR, United States; ^2^MD Anderson Cancer Center, Houston, TX, United States; ^3^Northeast Georgia Medical Center, Ganiesville, GA, United States; ^4^Department of Internal Medicine, Dr. NTR University of Health Sciences, Vijayawada, India; ^5^Indira Gandhi Medical College, Shimla, India; ^6^Genesis Cancer and Blood Institute, Little Rock, AR, United States

**Keywords:** lung cancer, screening, minimal residual disease, circulating tumor DNA, artificial intelligence

## Abstract

Non-small cell lung cancer (NSCLC) remains a leading cause of cancer mortality, with late-stage diagnosis contributing to poor survival. Circulating tumor DNA (ctDNA) has emerged as a non-invasive biomarker for screening, diagnosis, and monitoring, with limitations about sensitivity and specificity challenges. The integration of artificial intelligence (AI) offers a promising avenue to enhance ctDNA applications in NSCLC by improving mutation detection rates and sensitivities, refining minimal residual disease (MRD) predictions, enabling earlier detection of relapse, sometimes earlier than imaging, differentiating tumor vs. non-tumor derived signals to improve specificities. AI achieves 0.002% mutant allelic fraction detection, 94% relapse detection sensitivity, and 5.2-month lead time over imaging. This narrative review explores the role of ctDNA in NSCLC management, highlighting how AI amplifies its utility across screening, diagnosis, treatment evaluation, MRD detection, and disease surveillance while outlining key opportunities, challenges, and future directions.

## Introduction

The lung cancer incidence continues to increase with contrary change in survival, especially of stage IV disease (1, 2). Screening with low-dose computed tomography (LDCT) among certain high-risk individuals is the current recommendation, which still is underutilized, partly due to the risk of exposure to radiation and false positive results leading to invasive procedures causing potential harm (3). Tissue biopsies are the gold standard for diagnosis but carries procedural risks in 15%–30% of cases and may yield insufficient samples for molecular profiling (4). Moreover, traditional protein based biomarkers lack sensitivity and specificity for early detection and monitoring. The search for an exceptionally reliable, non-invasive biomarker is underway not only for lung cancer screening but also to guide initial diagnostics, precision treatments, predicting prognosis, and finding early relapse and actionable targets (5).

On the same lines, liquid biopsies broadly refer to the identification of one of the cell components derived from the tumor in the bodily fluids representing a viable surrogate of the tumor tissue (6). These components include circulating tumor cells (CTCs), circulating tumor DNA (ctDNA), cell-free tumor RNA (cfRNA), exosomes and tumor-educated platelets (TEP) (7). ctDNA has been widely studied and increasingly used noninvasive biomarker in addition to invasive tissue biopsy in many solid cancers including non-small cell lung cancer (NSCLC), leading its approval from United States Food and Drug Administration (U.S. FDA) especially in NSCLC (8). The integration of artificial intelligence (AI) and machine learning (ML) with ctDNA analysis further amplified its potential in revolutionizing its capabilities, from improving detection sensitivity to uncovering complex mutational patterns (9). In this review, we summarized the physiology of ctDNA and its application in NSCLC, including its role in screening, early diagnosis, individualized treatment, MRD detection, disease surveillance, treatment resistance and the future perspectives.

## Pathophysiology, isolation, and analysis of ctDNA

**Figure 1:**
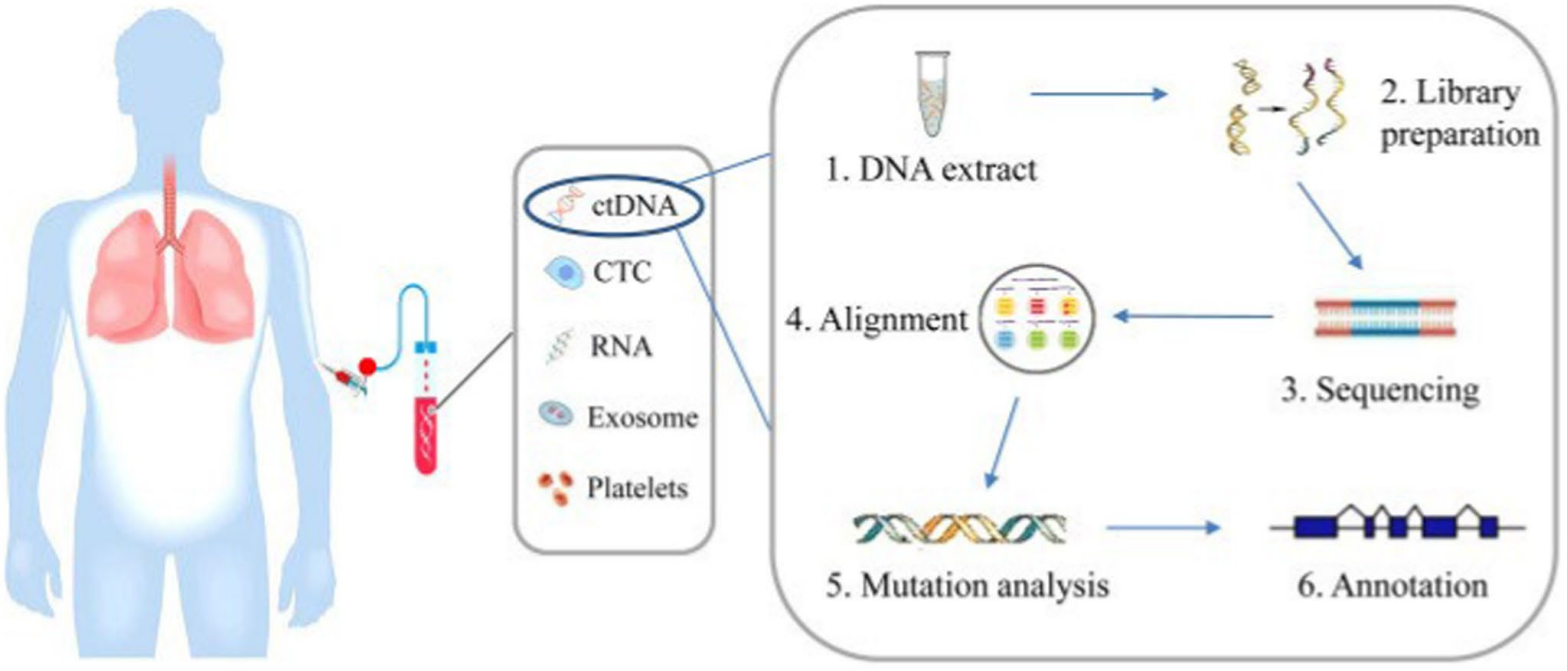
Blood is collected from patients and ctDNA is extracted from blood plasma and mutations can be analyzed by next generation sequencing involving a few steps, including DNA extraction, DNA library preparation, sequencing, sequence alignment, mutation annotation, and so on (58).

Mandel and Metais (10) first reported the presence of nucleic acids in blood circulation. Cell free DNA (cfDNA) can be found at low levels in the blood of healthy subjects and can be elevated in inflammatory, ischemic and pregnancy states. cfDNA was also reported to be found in other body fluids namely urine and spinal fluid (11–14). Circulating tumor DNA (ctDNA) is a type of cfDNA released from cancer cells due to a variety of processes. It ranges from 180 to 200 base pairs in length and has mutations pertaining to the tumor (15). The process of ctDNA isolation and analysis is illustrated in Figure 1.

Baseline ctDNA levels in NSCLC patients were shown to correlate with disease stage, burden, metabolism and higher cfDNA levels may independently predict poor progression free survival (PFS) and overall survival (OS) (16). Lower ctDNA levels at earlier stages of disease due to smaller disease burden poses a threat to disease detection and some studies recommended combining ctDNA with other cutting edge diagnostics like exosomal RNA to increase the sensitivity of detection (17). However, recent advancements in sequencing like digital polymerase chain reaction (PCR) and next-generation sequencing (NGS) will alleviate this problem with better mutant allelic fraction detections up to 0.05% (11). In addition to the recent technological advances, incorporating artificial intelligence (AI) and machine learning (ML) algorithms with these sequencing technologies to refine diagnostic accuracy by enhancing the detection of low-frequency mutations there by improving the sensitivity and specificity of ctDNA assays, particularly in early-stage NSCLC where tumor burden is minimal (18).

## ctDNA role in lung cancer screening

The United States preventive services task force (USPSTF) recommends LDCT for screening lung cancers among high-risk populations. If suspicious lesions were noted, standardized reporting was implemented using Lung reporting and data system (L-RADS) for further follow up and management. Using Lung-RADS criteria, ≥6 mm has been chosen as the lower limit of threshold for solid nodules to minimize false positive rates without affecting false negative rates (3). Lung cancer prognosis did not improve significantly in the last few decades even with advancement in therapeutics which was attributed to delay in diagnosis due to minimal early stage symptoms (19, 20). This is reflected in the tremendous difference of 5 year survival of 2 and 71% among patients with NSCLC diagnosed at stage IV disease and early stages, respectively (19). Therefore, early diagnosis of lung cancer patients might improve patient outcomes, thereby implicating the need for novel markers to aid in achieving the goal.

One of the major goals in oncology is detecting cancers at an early stage thereby being able to treat them with curative intent leading to better outcomes, which sparked the curiosity in finding a pan-screening test to be used in otherwise healthy subjects. Traditional protein-based biomarkers carry the risk of poor sensitivity and specificity there by limiting its routine use in earlier detection (21). Also, those markers were not available to all the available cancers, especially lung cancer. This led to the idea of using ctDNA as a potential marker for identifying cancers at an earlier stage. Like protein-based markers, cancer cells are thought to secrete actively or passively ctDNA into the circulation which help us not only to identify the tumor type but also targetable mutations to guide better therapeutics.

The Circulating Cell-free Genome Atlas study (CCGA) by GRAIL, used a multi-cancer early detection test utilizing cfDNA and AI and reported an overall sensitivity of 51.5% (Range: 14.5–92.2%) in detecting cancers with sensitivity directly proportional to the disease stage (22). The study used AI and ML by employing targeted methylation-based sequencing of cfDNA, using a ML classifier to differentiate tumor derived signals from non-tumor derived ones which improved specificity (reported at 99%) to enable cancer type identification. Similarly, Cohen et al. (23) reported sensitivities ranging from 69 to 98% for the detection of five cancer types (ovary, liver, stomach, pancreas, and esophagus) with changes depending on cancer stage and type. Sensitivities were highest in higher stages and solid tumors of ovarian or liver origin and least in earlier stages and breast cancer and also increased when combined with imaging modalities like PET-CT scan and standard protein biomarkers for some of these cancers (23–25). For lung cancer, the probability of detection was reported as 75% and both studies reported an overall specificity of 99%. The sensitivity, which is one of the crucial aspects of a screening test, is lower especially in earlier stages, which might be a drawback for some cancers but useful in other cancers like lung or liver where the earlier stages have better sensitivities (26).

Recent advancements in ctDNA-based screening have significantly improved early detection of non-small cell lung cancer (NSCLC). Mathios et al. (1) utilized cfDNA fragmentome analysis with ML to achieve 75% sensitivity for Stage I–II NSCLC and 95% specificity, offering a novel approach to distinguish tumor-derived signals from non-tumor cfDNA. Similarly, Yin et al. (27) demonstrated that combining ctDNA with protein biomarkers (CEA, SqCC, CYFRA21-1) increased sensitivity to 86.4% for early-stage NSCLC, highlighting the potential of multi-analyte approaches. Additionally, Tan et al. (28) reported a 65% sensitivity for Stage I–II NSCLC using ultradeep sequencing, with a specificity of 98.5%, addressing challenges like low mutant allele fractions and clonal hematopoiesis of indeterminate potential (CHIP) through tumor-informed sequencing. These studies collectively underscore the evolving role of advanced sequencing, bioinformatics, and multi-biomarker strategies in enhancing lung cancer screening accuracy for high-risk populations.

Although a direct comparison with standard protein biomarkers was not performed, the available results enlighten us for a possible new horizon in the future with better emerging technologies for improving the sensitivities (Table 1). On the contrary, Pons-Belda et al. extrapolated using the current available data from Ct-DNA diagnostic methods and reported that the current detection methods will identify tumors of size 10-15 mm in diameter. Tumors below this size lead to incredibly low mutant allelic fraction about 0.01% which will reduce the sensitivity rendering the use of this technology implausible for screening methods (29).

**Table 1:** Comparison of multi-analyte blood tests for lung cancer screening.

Study	Methods	Key findings	Lung cancer specific findings
Cohen et al., 2018 (23)	Combined circulating tumor DNA (ctDNA) + protein biomarkers. Analyzed 1,005 cancer patients (non-metastatic, eight types). Assessed specificity in 812 healthy individuals.	Overall sensitivity: 70% across eight cancer types. Specificity >99% in healthy individuals. Cancer location correctly identified in 83% of positive cases.	Lung cancer sensitivity: 59%. Detected 56% of Stage I lung cancers. Sensitivity improved in later stages.
Klein et al., 2021 (22)	Evaluated MCED test in 4,077 participants (2,823 with cancer, 1,254 without). Included 50 + cancer types, analyzed across different stages. Real-world validation study.	Overall sensitivity: 51.5% (varied by stage). Sensitivity increased with stage: 16.8% (Stage I), 40.4% (Stage II), 77.0% (Stage III), 90.1% (Stage IV). Specificity: 99.5%. Cancer signal origin identified correctly in 88.7% of cases.	Lung cancer sensitivity: 41% overall. Lower detection rate for early-stage lung cancer. Higher detection in later stages (Stage III-IV).
Mathios et al., 2021 (1)	cfDNA fragmentome analysis machine learning. 200 NSCLC patients (Stage I–IV).	Sensitivity 75% for Stage I–II; specificity 95%; tumor-specific mutations identified.	Lung cancer sensitivity 75% for early stages; improved detection with fragmentome-based approach.
Yin et al., 2022 (27)	Combined ctDNA and protein biomarkers (CEA, SqCC, CYFRA21-1); multi-gene panel. 300 NSCLC patients.	Sensitivity 86.4% for Stage I–II; specificity 97%.	Lung cancer sensitivity 86.4% when combining ctDNA with protein markers; higher detection in early stages.
Tan et al., 2024 (28)	Ultradeep sequencing of cfDNA; tumor-informed approach. 500 NSCLC patients (Stage I–IV).	Sensitivity 65% for Stage I–II; specificity 98.5%; reduced false positives via CHIP filtering.	Lung cancer sensitivity 65% for early stages; improved detection with tumor-informed sequencing.

Another important setback with using ctDNA as a screening test is the presence of clonal hematopoiesis of indeterminate potential (CHIP), a benign condition usually noted in healthy people of older age that causes the release of mutant DNA into the circulation thereby causing false positive rates. Although CHIP-based mutations can be differentiated from the real mutations from malignancy using advanced sequencing steps, this poses a time consuming and costly process, limiting its role as a lung cancer screening tool (30).

## ctDNA role in primary diagnosis and treatment evaluation

In addition to traditional TNM staging, NSCLC classification evolved to other subtypes based on the detection of various genetic mutations and subsequent treatment with concomitant targeted therapies, leading to better outcomes (31). Tissue biopsy remains to be the gold standard in diagnosing lung cancer. More commonly tested genetic mutations in NSCLC include *EGFR* mutations, *ALK* rearrangements, and *ROS1* fusions in addition to *MET*, *RET*, *BRAF*, *HER2*, and *NTRK1*. Plasma based conventional tumor markers like carcinoembryonic antigen (CEA), neuron specific enolase (NSE), etc., were of limited utility in aiding primary diagnosis (32). The intrinsic features of ctDNA seems to be an attractive non-invasive method to help in primary diagnosis. It emerged as an equally effective alternative noninvasive detection method for aiding in primary diagnosis and detecting resistance mutations compared to more invasive biopsy testing and may be used with other available biomarkers to enhance the quality of primary diagnosis results (33).

The quest to replace high risk, invasive tissue biopsy to low risk, least invasive, patient tolerated liquid biopsy is ongoing. Even though earlier small scale studies showed discordance between the somatic variations among tissue and plasma samples, recently performed large scale and appropriately designed studies reported better concordance between the samples, thereby encouraging the use of ctDNA in primary diagnostics (34, 35). It was suggested by international association for the study of lung cancer (IASLC) that ctDNA implementation might improve the patient outcome and it should be routinely implemented in clinical practices (36). On the same lines, data from the ENSURE, AURA phase 2 extension cohort and AURA 2 studies lead to approval of Cobas *EGFR* Mutation Test v2 (Roche Molecular Diagnostics, Pleasanton, CA) by U.S. FDA to detect specific mutations (exon 19 deletion or exon 21 [L858R] substitution) in patients’ blood with NSCLC to determine candidates for treatment with erlotinib as well as in patients with T790M mutations who would benefit from Osimertinib (37–39). European agency also approved another ctDNA test (Thera screen EGFR RGQ PCR Kit, Qiagen, Valencia, CA) to detect EGFR mutations when tumor tissue is insufficient (40).

All these studies supporting the use of ctDNA in aiding primary diagnosis had an unquestionable specificity of around 99%. However, the negative predictive value is low, which will lead to false negative results and should always be followed by gold standard tissue-based testing. Patients who progress on Osimertinib should always checked for EGFR-C797S, and other rare genetic alterations (BRAF-V600, KRAS, HER2 and MET) and ctDNA will be an extremely useful least invasive intervention with a quick turnaround time, allowing targeting additional alterations (41–43).

In addition to EGFR related mutations, ctDNA can be used to detect other genetic changes too. In the largest prospective cfDNA study by Leighl et al. (33), among previously non treated metastatic NSCLC, liquid biopsies using cfDNA identified FDA approved markers (i.e., *ALK*, *BRAF*, *EGFR*, and *ROS1*) in addition to other alterations (ERBB2, RET, MET amplifications and exon 14 skipping) at a high concordance rate with tissue biopsies reaching up to 98% among FDA approved ones signifying its role in effective genotyping thereby precisely designing therapies for patients.

Dong et al. (44) demonstrated that ctDNA-guided de-escalation of tyrosine kinase inhibitors in advanced NSCLC achieved complete remission in 60% of patients, with a 95% concordance rate for actionable mutations (EGFR, ALK, ROS1). Provencio et al. (45) showed that ctDNA clearance post-neoadjuvant nivolumab plus chemotherapy in Stage IIIA NSCLC predicted improved 5-year overall survival (HR 0.35, *p* = 0.002), highlighting its utility in immunotherapy settings. Longitudinal ctDNA monitoring is increasingly vital for detecting treatment resistance. Ding et al. found that an early ctDNA nadir within 6 weeks of chemoimmunotherapy predicted better progression-free survival and overall survival in metastatic NSCLC (HR 2.8, *p* < 0.001), with emergent mutations (e.g., KRAS, MET) indicating resistance. These advancements underscore ctDNA’s potential to guide adaptive therapy (46).

## ctDNA in detecting MRD

The pursuit to identify early relapse post curative treatment in any malignancy is a matter of high regard. Current practices involve relying on clinical, radiological, and plasma-based tumor markers to identify early relapse in NSCLC patients treated with curative intent. The use of ctDNA in detecting minimal residual disease (MRD) among NSCLC patients treated with curative intent at their various post-treatment time points was forthcoming. It has been already shown to identify early relapse in the aforementioned group in various small retrospective studies, and their clinical validation through large prospective trials is ongoing.

Chaudhuri et al. first observed the ctDNA levels in a group of unresectable NSCLC patients varying from Stages I–III. They reported that 17 out of 32 patients, with detectable ctDNA within 4 months of completed treatment had lower freedom from progression (FFP) and disease specific survival than those with undetectable ctDNA in the same time point. On the same lines, all 17 with MRD +ve but only 1 out of 15 MRD −ve of them relapsed in the same follow-up period. The study utilized Cancer Personalized Profiling by deep sequencing (CAPP-Seq), a targeted Next generation sequencing (NGS) approach focusing on recurrent NSCLC mutations to achieve high sensitivity (down to 0.002% mutant allele fraction) but potentially missing rare mutations (47).

Similarly, Modling et al., conducted a retrospective study on unresectable stage IIB-IIIB NSCLC patients who received chemoradiotherapy (CRT) initially and further stratified into consolidation with immune checkpoint inhibitor (ICI) and no consolidation group. Plasma samples for ctDNA were checked pre-CRT, post-CRT and median of 11 weeks into ICI therapy. Among no consolidation cohorts, 1 out of 12 post CRT MRD −ve and all 17 of post CRT MRD +ve relapsed in 12 months of follow up. Among the consolidation group, increased freedom from progression (median 22 months vs. 5 months) was observed among MRD +ve post CRT with decreasing ctDNA levels pre-ICI to early on ICI than increasing ct DNA levels during the same time (48).

Chen et al., conducted a prospective study in November 2016 among NSCLC patients with stages I-III who underwent surgical resection for curative intent and ctDNA measured at various time points including (1) immediately before surgery, (2) 5 min, 30 min, and 2 h after surgery, and (3) 1, 3, and 30 days after surgery. Based on the study results, they concluded that the median half-life of ctDNA was 35 min and its longer in patients with positive ctDNA 1–30 days post-surgery than those with undetectable levels in the same period. The authors also suggested measuring ctDNA post operatively as early as 3 days can accurately prognosticate survival by predicting the relapse risk (49).

Another prospective study by Abbosh et al. reported the detection of ctDNA levels at or before clinical relapse among 82% of Stage IA-IIIB NSCLC patients who underwent surgical resection. However, in patients who remained relapse-free during a median follow up of 1,184 days, ctDNA was detected at only one of the 199 timepoints (50).

Recent studies have strengthened ctDNA’s role in detecting minimal residual disease (MRD) in NSCLC. Gale et al. (51) validated ctDNA in 88 early-stage NSCLC patients post-treatment, achieving 90% sensitivity and 95% specificity for MRD detection, predicting relapse 4.8 months before radiographic recurrence. Similarly, Isbell et al. (52) used ultrasensitive sequencing to detect ctDNA in early-stage NSCLC, reporting 92% sensitivity for MRD post-surgery and a 5–7-month lead time, with tumor-informed panels reducing CHIP-related false positives. The LUNGCA-1 study further confirmed ctDNA’s utility, finding that persistent ctDNA at 3–7 days post-surgery predicted relapse with 92% sensitivity and a 6.1-month lead time in 330 patients (53).

The aforementioned studies in surgical resection patients used targeted deep sequencing with limited panels to detect ctDNA. This is evident in a study by Abbosh et al., where 10 patients during clinical follow up were diagnosed with non-lung primary malignancy but their ctDNA did not identify them. Zviran et al., used tumor-based whole genome sequencing (WGS) to detect MRD in the plasma samples at 2.5 weeks before and after surgery among NSCLC patients. On a median follow-up of 18 months, 50% of post-surgery MRD positive patients relapsed while 100% of MRD negative group non-relapsed (54).

All the above studies emphasize the role of ctDNA in MRD detection among early-stage NSCLC patients treated with curative intent by providing insights into the long-term prognosis (Table 2). There is potential for AI models to refine the sensitivity of targeted sequencing panels and integrate longitudinal ctDNA data with clinical variables to predict relapse earlier and with greater accuracy. However, challenges include optimizing AI algorithms to account for tumor heterogeneity and reducing false negatives, particularly when using limited gene panels (55). In the study by Chaudhuri et al., ctDNA predicted relapse in about three-fourths of patients a median of 5.2 months earlier than radiological relapse, thereby providing more time to change therapies for better outcomes. In addition, they reported 100% sensitivity and specificity of ctDNA in detecting recurrences using an ever-positive vs. never-positive approach. However, the sensitivity of MRD detection in post-surgical patients is not optimal, except in Zviran et al., where tumor-based WGS was used. Based on the above evidence, multiple prospective clinical trials are ongoing with the possible outcome of routinely using ctDNA for MRD and disease surveillance, thereby improving outcomes and alleviating toxicities through precision treatment (56).

**Table 2:** Summary of studies evaluating circulating tumor DNA (ctDNA) as a biomarker for minimal residual disease (MRD) detection in lung cancer.

Study	Study design and population	Methodology	Key findings
Chaudhuri et al., 2017 (47)	Retrospective cohort; 40 patients with localized lung cancer (stages I–III) treated with curative intent (surgery/radiotherapy).	Targeted NGS of ctDNA using CAPP-Seq; pre-and post-treatment plasma samples analyzed.	ctDNA detected MRD in 94% of relapsing patients, median lead time 5.2 months before radiographic recurrence. Specificity 96% for non-relapsers.
Moding et al., 2020 (48)	Prospective cohort; 65 patients with unresectable stage III NSCLC post-chemoradiotherapy, treated with anti-PD-L1 (durvalumab).	CAPP-Seq for ctDNA quantification; serial plasma sampling pre-and post-immunotherapy; correlated with PFS and OS.	Undetectable ctDNA post-chemoradiotherapy linked to better PFS (HR 0.29, *p* = 0.004); ctDNA clearance during immunotherapy predicted benefit (HR 0.13, *p* = 0.0003).
Chen et al., 2019 (49)	Prospective cohort; 36 NSCLC patients undergoing curative-intent surgery (stages I-IIIA).	Targeted NGS panel (168 genes) for ctDNA; plasma collected pre-op, 3 days post-op, and up to 120 days post-op.	ctDNA half-life ~35 min post-surgery; persistent ctDNA at 3 days post-op correlated with recurrence (HR 11.14, *p* < 0.001). Sensitivity 90% at 120 days.
Abbosh, 2017 (50)	Prospective cohort (TRACERx); 100 patients with early-stage NSCLC (stages IA-IIIA) undergoing surgery.	Multiregion whole-exome sequencing of tumors; targeted NGS of ctDNA for clonal/subclonal mutations; longitudinal sampling.	ctDNA reflected tumor phylogeny; subclonal mutations predicted relapse (*p* = 0.001). 94% of relapsing patients had detectable pre-op ctDNA vs. 33% in non-relapsers.
Zviran, 2020 (54)	Mixed cohort; 137 patients (including lung cancer subset) post-treatment; validated in 208 additional samples.	MRDetect: genome-wide cfDNA mutation integration via WGS; signal-to-noise optimization for ultra-sensitive detection.	Sensitivity of 10^−5^; in lung cancer, MRD detection preceded relapse by up to 200 days (*p* < 0.001). False-positive rate < 2%.
Gale et al., 2022 (51)	Prospective cohort; 88 early-stage NSCLC patients (Stage I–IIIA) post-treatment.	Tumor-informed NGS; ctDNA measured post-treatment and longitudinally.	90% sensitivity, 95% specificity for MRD; 4.8-month lead time before relapse.
Isbell et al., 2024 (52)	Prospective cohort; early-stage NSCLC patients post-surgery.	Ultrasensitive sequencing; tumor-informed panels; ctDNA measured post-op.	92% sensitivity for MRD; 5–7-month lead time; reduced CHIP false positives.
Xia et al., 2022 (53)	Prospective cohort; 330 NSCLC patients (Stage I–IIIA) post-surgery.	168-gene NGS panel; ctDNA measured 3–7 days post-op and longitudinally.	Persistent ctDNA at 3–7 days predicted relapse (92% sensitivity, 90% specificity); 6.1-month lead time.

## Future perspectives

The field of ctDNA research is rapidly evolving, with ongoing advancements in sequencing technologies, bioinformatics, and multi-analyte approaches. The integration of AI and ML into ctDNA analysis holds promise for improving the accuracy and efficiency of mutation detection. Additionally, the development of multi-cancer early detection (MCED) tests that combine ctDNA with other biomarkers, such as exosomal RNA and protein markers, could enhance the sensitivity and specificity of cancer screening.

AI is significantly transforming ctDNA analysis for NSCLC. For instance, Gale et al. employed AI bioinformatics to achieve 90% sensitivity in detecting MRD and 95% specificity in identifying resistance mutations like EGFR T790M. Similarly, Mathios et al. used ML on cfDNA fragmentomes, reaching 75% sensitivity for early-stage NSCLC. Furthermore, Kris et al. (57) utilized AI to analyze ctDNA changes over time in neoadjuvant atezolizumab patients, predicting relapse with 85% accuracy. These studies demonstrate AI’s capability to combine various data types and account for the diverse nature of tumors. However, challenges such as validating AI models across diverse cohorts, managing computational complexity, and ensuring cost-effectiveness must be addressed to realize these future directions fully.

## Conclusion

Circulating tumor DNA (ctDNA) has emerged as a transformative tool in the management of non-small cell lung cancer (NSCLC), offering a non-invasive, dynamic, and comprehensive approach to cancer detection and monitoring. From screening and diagnosis to treatment selection and MRD detection, ctDNA has demonstrated significant potential to improve patient outcomes and redefine the standard of care in NSCLC. AI integration enhances its utility by achieving ultra-low detection limits and improved specificity.

While challenges remain, including the need for improved sensitivity in early-stage disease, clonal hematopoiesis interference and the integration of ctDNA into clinical workflows, ongoing research and technological advancements are expected to address these limitations. Clinicians should integrate ctDNA testing into routine practice, particularly for most common actionable mutations like EGFR, ALK and for MRD monitoring post-curative treatment. The continued evolution of ctDNA-based technologies, coupled with the integration of multi-analyte approaches and AI-driven analysis, holds promise for revolutionizing cancer care and achieving the ultimate goal of precision oncology.
